# Mr. Mayo's Introductory Lecture to the Medical Classes in King's College, London

**Published:** 1835-01-01

**Authors:** 


An Introductory Lecture to the Medical Classes in King's College, London. By *Herbert Mayo*, F.R.S. Professor of Anatomy, &c. &c. &c. 8vo. stitched, pp. 28. London, 1834.

We know not whether the love of fame or the hope of profit induces lecturers to give their introductory discourses to the world. Perhaps the former feeling usually predominates, and the memory of the cheer that burst upon the orator as he blushed and bowed, pursues him in his triumphal homeward march, and warms him to daring deeds of print. However this may be, it is certain that many a good twenty-pounds is ungrudgingly devoted to the propagation of lectures introductory and valedictory. We will not say that the sordid calculations of mercenary minds would be generally satisfied by the returns. Yet there cannot be a doubt that the convenient pamphlet is frequently applied to necessary purposes, and the philosophic butterman or trunkmaker has often the advantage of conversing at his leisure with learned and eloquent professors.

We are induced to notice Mr. Mayo's lecture, because it adverts to the engrossing subject of general and medical education. We agree with Mr. Mayo that in this the most important improvements have been made, and that we cannot yet fully appreciate their consequences. But this is so obvious as to constitute a truism, and we pass to an interesting question—the comparative utility of classical and mathematical attainments.

Mr. Mayo supposes, or seems to suppose, that the tendency of the day is to disparage the cultivation of classical literature, to despise the monuments of genius and taste contained in the languages of Greece and Rome.

"The acquirements," says he, "to which I am disposed to assign the first place, are a knowledge of the classical languages of antiquity, and an acquaintance with classical literature. It is the tone with some at the present day to undervalue these attainments, which hitherto have constituted the basis of what has been emphatically called a *liberal education*, to consider the reverence entertained by many for them, as a prejudice which is on the eve of being dispelled, and to recommend in their place the adoption of studies of more *practical* utility. For my own part, I am willing to argue the value of these studies, on the ground of their usefulness alone." 7.

We doubt the reality of Mr. Mayo's fears, and we question if any, whose opinion is regarded or worthy of regard, would desire to substitute any studies whatever for those of a classical description. But we know there is a feeling, a strong, and we believe a most judicious, feeling against the abuse of classical instruction—against the ridiculous and monkish system pursued at many of our public schools. It is notorious that, at many of those establishments, the boy is crammed with Latin and with Greek, to the absolute exclusion of other information. Educated at one of the great public schools ourselves, we can feelingly depict and deplore the monstrous and insane inadequacy of their method to effect the real objects of tuition. The generality of the boys so educated acquire not much of the two dead languages they study, and nothing of that knowledge which lives and breathes.

The utilitarians of the present day have published a crusade against the continuance of this antique folly—have exposed and denounced the exclusive study of the classics—and have recommended, what all men of common sense must approve, the comprehension of the modern European languages, French, German, and Italian, with the sober and exact studies of geography, arithmetic, and mathematics, in the general scheme of youthful education. At the schools where the middle classes are instructed, the description of study is as wide as this. If the aristocracy will persist in debarring their sons and their connexions from the useful knowledge of the useful ranks, it requires little foresight to descry the changes which society will undergo. The hard necessities of an industrious nation will refuse to respect what is merely ornamental.

It may be delightful, but it is not permitted us, to pursue through the fair regions of ancient taste and genius, the charms and the advantages of classic lore. The mind not imbued with its beauties and its fictions is no better than an untilled waste; it may be a strong and a hardy soil—but the fairest flowers and the richest herbage find no life nor shelter there.

Monumens du Genie, heureuses fictions,
Vous savez animer l'air, la terre, et les mers;
Vous embellissex l'Univers.

An aqcuaintance with the more accessible treasures of antiquity is indispensable alike for the gentleman and man of taste. Without it, we could not mix with elegant society, nor be received as the associate of men of cultivated understandings. To borrow another passage from that eloquent "Apology for Fiction," which proves while it defends, the classical predilections of the sovereign wit of France:—

On chérira toujours les erreurs de la Gréce,
Toujours Ovide charmera;
Si nos peuples nouveaux sont Chrétiens à la messe,
Ils sont Payens à l'Opera.

Mr. Mayo has not forgotten that classical learning is possessed of immediate and appreciable utility.

"As language is the instrument of thought, the basis of intellectual education should be the mastery of language. *One* language, it is true, rich and various as our own, should be sufficient to contain and express each element of human learning and thought. But it happens, that our native language, a compound of several tongues, is borrowed to so great an extent from the Latin, that its force and meaning, nay its construction, cannot be perfectly mastered, without a knowledge of that language at the least. I do not advert to terms of science, when asserting that the English language is imperfectly understood by the mere English student. These, derived principally from the Greek, it is evident must appear to *him* a barbarous and difficult, as well as an unmeaning nomenclature. But almost all our general and abstract terms have a Latin etymology; and their spirit cannot be perfectly caught, unless the language in which they were formed is known, in which the transition was made from sensible images to general expressions." 8.

We need not pursue the subject any further. We believe that no reasonable, certainly no well-informed, writer has attempted to abolish classical instruction. But in our public schools, and, perhaps, in some of our private seminaries, it has been most unwisely erected into the beginning and the end of the knowledge adapted and conveyed to youth. This is an abuse that the common sense of the present day will tolerate no longer.

Mr. Mayo has appeared the enthusiastic champion of the classics. His defence of mathematics is cold and dull. It looks, in pugilistic language, like a *cross*.

" It admits of a question, to what extent mathematical studies should form a part of medical education. Pure and mixed mathematics, the method of analysis, and its application to natural philosophy, constitute indeed the fairest triumph of human reason. The greatest intellect, that ever rose above humanity, found in these studies, and in the order of nature which they served to elucidate, the single subject worthy its genius. But mathematical studies have no direct application to medicine ; and if a time is to come, when physiology shall rank among the exact sciences, that period is certainly very distant. To the medical student, mathematical studies may seem therefore to have little value, except as an exercise and discipline of the understanding. It has been thought by some that their value even in this respect is equivocal ; and it has been argued, not without plausibility, that the habit of looking for demonstration unfits the intellect for weighing moral truth ; and that in proportion as the mind is accustomed to absolute certainty in every step of reasoning, in so far does its power of estimating probability suffer by disuse ; while a distaste is acquired for any study, to which demonstration does not apply." 11.

The extent to which mathematical studies should be cultivated may probably admit of question. But no question can exist with respect to the advantage of some, we say much, mathematical knowledge. The anticipation of too great a disposition towards exactness on the part of surgeons and physicians, is more calculated, we think, to amuse than to alarm. One might almost reply to the apprehensive gentlemen who dread the application of analysis to medicine, in the consolatory tone of the captain to his passenger, who asked, " if there was any fear"—" Plenty of fear, Ma'am, but no danger."

There is indeed one sufficient answer to those whose timidity or prudence would set limits to the acquisition of this or that species of knowledge. Those limits are always too easily attained, and the spur is in general more necessary than the curb. The period assigned to elementary and general study is commonly too brief to procure what is desirable, seldom sufficient to allow the most able and zealous neophyte to pursue coy Learning to a dangerous distance. We will venture to assure Mr. Mayo and others, that they need not be afraid of the youth designed for business or professions, enjoying leisure or evincing the desire to proceed too deeply into classics, mathematics, or any other mine of human knowledge. Let but the system of education be judicious, and there cannot be too much of it. In that system exact studies should form a very large component part.

We know not that there remain any general questions, on which we may introduce the lecturer or ourselves. Yet we cannot refrain from giving utterance to a remark not more complimentary than true. The lecture before us is a fair example of the cultivated taste and the acute observation, begot from the acquirements which it eloquently advocates.

The medical portion of the lecture is respectable—the physiological, interesting though brief. Utilitarians in journalism, we cannot allow this brief article to depart without ballasting its light and general argosy, with a keel of substantial inflammation.

Mr. Mayo's description of the vital phenomena in the living egg is a beautiful abstraction of fact and observation. He takes it, and " looks where life is sleeping, at that which is to form it."

"We may follow," says he, "in the living egg, the changes recently discovered to take place in it, new in our philosophy, which begin when the influence of one chemical agent is made to tell. There is in the egg, resting immediately upon the yelk, a little circular disc of the thinnest membrane. Its diameter is no greater than one-sixth of an inch. It is divided into an outer zone, and an inner, clearer, and more transparent part, called the colliquamentum. This disc of membrane is all the trace of the future chick. It is called the cicatricula, germ spot, germinal membrane, or blastoderma.

When the egg is exposed to the proper temperature, the process of development begins. Six hours after incubation has commenced, a small dark line may, with the aid of a magnifying glass, be discovered towards the centre of the transparent area. This line, which is called the primitive trace, is swollen at one extremity, and is placed in the direction of the transverse axis of the egg. The rounded end is towards the left, when the small end of the egg is turned from us.

Towards the twelfth or fourteenth hour of incubation, the germinal membrane expands itself into a larger area; and at the same time acquiring thickness, it separates into two layers. Of these, the outer is called the serous layer. The inner, in contact with the yelk, is termed the mucous. The two layers appear each to consist of coherent granules. After these two layers have appeared, a third is formed in the interval between them: this is called the vascular layer; it has a more intimate connection with the inner or mucous layer than with the outer or serous.

The primitive trace is placed in the outer or serous layer. About the eighteenth hour, two ridges are raised from the serous layer, one on either side of the primitive trace, inclosing a furrow. This furrow soon becomes closed at the swollen extremity of the primitive trace, at which part it is of a pyriform shape, being wider here than at any other part, Thus the serous layer, thickening round the primitive trace, shapes itself into the figure of a body and trunk; having absorbed from the albumen the material necessary for its growth.

In the canal formed as it has been described, a semi-fluid matter appears, which, on its acquiring more consistency, becomes the rudiment of the spinal marrow. The same matter collects in the pyriform chamb-r at the top into three bladders, which are the rudiment of the brain. As the brain and spinal marrow, so are the bones, nerves, muscles, and tegumentary systems of the body, developed in the outer or serous layer.

The inner or mucous layer, folding itself while it expands, forms the rudiment of the alimentary canal.

The middle or vascular layer originates the heart and blood-vessels; and, conjointly with the mucous layer, the glandular system and the lungs." 18.

We cannot conclude without expressing the pleasure we have felt in glancing over this published lecture. Trifle as it is, we lay it down with a heightened opinion of the intelligence and attainments of its author.

